# Supplementary material for: Environmental uncertainty shapes human effort learning
Source: PLoS Biol. 2026 May 7;24(5):e3003791. doi: 10.1371/journal.pbio.3003791 (PMC13175491; doi:10.1371/journal.pbio.3003791)
Supplement: S1 Text — A full description of the results for all supplementary figures. (DOCX) [file pbio.3003791.s002.docx]

# **Supplementary Information**

**Environmental Uncertainty Shapes Human Effort Learning**

Rong Bi ^1,2*^, Jan Grohn ^2,3^, Patricia Lockwood ^4,5^, Miriam C. Klein-Flügge ^1,2,3&*^, Lilian Weber ^1,2,6&*^

^&^Shared Senior Authors

^*^Corresponding authors: Rong Bi ([rong.bi@psych.ox.ac.uk](mailto:rong.bi@psych.ox.ac.uk)) and Miriam C. Klein-Flügge ([miriam.klein-flugge@psy.ox.ac.uk](mailto:miriam.klein-flugge@psy.ox.ac.uk)) and Lilian Weber ([lilian.weber@psych.ox.ac.uk](mailto:lilian.weber@psych.ox.ac.uk))

<https://doi.org/10.1371/journal.pbio.3003791>

# Supplementary results

## Force traces show evidence of effort learning (S2 Fig)

To check whether most change trials exhibited the same pattern as in **Fig 2B** and **Fig 2C**, we scaled required effort to 1 in each trial and averaged force traces across all participants. As shown in the average force traces, participants’ priors overshot the required effort in downwards change trials and undershot it in upwards change trials (**S2A Fig**). These errors were reduced in the subsequent trial. Consistently, example downwards change trials, upwards change trials and their subsequent trials from randomly selected trials in 10 participants showed the same pattern (**S2B Fig**).

## The estimated prior is not driven by sensorimotor feedback (S3 Fig)

As our measure of prior is defined at the initial plateau of the force trace, real-time sensorimotor feedback may have been integrated into the estimated prior. However, such integration is likely limited given the relatively short duration of the prior stage (M = 230.32 ms, SD = 25.11 ms) compared to the subsequent adjustment stage (M = 475.37 ms, SD = 76.58), where most feedback-driven adjustments are likely to occur.

In an ideal case, where no feedback is integrated, the deviation of the prior on trial t from the required effort on trial t would perfectly scale with jump size, i.e. the unpredictable difference in required effort levels between trial *t* and trial *t-1*. If this was the case, the prior would reflect only previous expectations but not current requirements. This can be most conservatively investigated at change trials, where the prior is off and should be updated rapidly, and where sensorimotor feedback should most clearly indicate this deviation.

To quantify the influence of sensorimotor feedback on the prior, we therefore examined how the difference between the prior on trial *t* and the effort requirement on trial *t* varied with jump size for change trials (i.e., difference in required effort from trial *t-1* to trial *t*, ignoring noise). We found that the difference between prior and current effort scaled with jump size (**S3 Fig**), and that the observed slope of -0.779 (red line) was only slightly flatter than the ideal slope (i.e., slope = -1; black line). This deviation could indicate a small influence of sensorimotor feedback. It was more pronounced for downwards and smaller for upwards change trials, potentially reflecting an energy-conserving strategy (for negative down-ward changes, naturally the prior was at a higher effort level). Thus, these results suggest that our estimated prior primarily reflects expectation-based beliefs rather than feedback-driven integration.

## The force initiation is not confounded by long-term fatigue (S4 Fig)

As low noise blocks were always presented before high noise blocks, observed noise effects (longer start RTs in high noise compared to low noise blocks) might be confounded by long-term fatigue (longer start RTs in later compared to earlier blocks). To rule out such fatigue effects, we conducted two control analyses that examined whether start RTs increased from the first low noise block group (block1+2) to the second block group (block3+4) (fatigue effect), from the first high noise block group (block5+6) to the second block group (block7+8) (fatigue effect), or only selectively from low to high noise blocks (noise effect). Additionally, we tested whether start RTs would slow down more with increased trial number in later compared to earlier blocks, which would also be due to fatigue effects. Our analyses below only find significant noise but no significant long-term fatigue effects.

First, we repeated the regression explaining start RTs shown in main **Fig 3A**, but this time we added additional control regressors: *block group difference* between the second block group (1) and the first block group (-1) (block3+4 – block1+2; all low noise blocks), *block group difference* between the fourth block group (1) and the third block group (-1) (block7+8 – block5+6; all high noise blocks), *block-wise noise difference* between the high (1) and low (-1) noise blocks (high noise – low noise) and *trial number* (trial 1-96) within each block group (**S4A Fig**). The influence of block group difference on start RTs did not significantly differ in either the low noise blocks (block3+4 – block1+2: *t*(27) = 0.532, *P* = 0.5994) or high noise blocks (block7+8 – block5+6: *t*(27) = 1.404, *P* = 0.1718). However, the block-wise noise difference had a significant effect on start RTs (*t*(27) = 4.135, *P* = 0.0003), showing a longer start RTs with increased noise difference. Also, start RTs increased with trial number per block group (*t*(27) = 4.775, *P* < 0.0001). Taken together, these results are consistent with a step-change in start RTs that is driven by the noise in the environment, and less consistent with the idea that average start RTs gradually increased over time due to fatigue (blocks or trials).

Second, we fit separate linear regression models to start RTs in each block group, explaining start RT with a regressor capturing the trial number (trial 1-96). We compared the resulting slopes across block groups using a one-way repeated-measures ANOVA. The slopes did not differ significantly between block groups (*F*(3,81) = 1.937, *P* = 0.1468; **S4B Fig**). This control analysis also suggests that steeper increases in fatigue later in the experiment cannot explain the noise effects reported in the main manuscript.

## Effort learning is not influenced by motor noise (S5 Fig)

To evaluate whether motor noise contributes to learning during effort production, we examined the contribution of motor noise at both the trial-wise and subject-wise levels.

We first quantified trial-wise motor noise for each participant. Motor noise was defined as the standard deviation of the force trace during the stable period. The onset (green circle) of this window had already been obtained (**S5A Fig**). To determine the offset (red square), (1) we smoothed the derivative of the raw force trace (smoothing kernel: 40 samples); (2) we then computed the derivative of the smoothed force trace; (3) finally, we identified the latest time point in the force trace where both the smoothed force exceeded 0.1 and its derivative surpassed a threshold of -0.0001. All trials were subsequently visually inspected blind to condition, and the offset was manually corrected if necessary. This only affected 0.31% of trials. The standard deviation of the force trace within this period (width of grey shading) was then used as the trial-wise estimate of motor noise.

To validate our measure of motor noise before examining relationships with learning, we tested whether motor noise scales with required effort. For each participant, we fitted a linear regression to motor noise as a function of required effort level. Across participants, the slope was significantly greater than zero (*t(*27) = 16.413, *P* < 0.0001; **S5B Fig**), suggesting that motor noise increases with higher effort.

To assess the contribution of motor noise to trial-wise learning, we therefore fitted a linear regression, modelling the absolute prior update in the current trial as a function of the motor noise and effort requirements in the previous trial. The results show that prior updating was significantly influenced by the previous trial’s experienced effort (*t*(27) = 5.563, *P* < 0.0001), but not by motor noise (*t*(27) = -0.177, *P* = 0.8606) (**S5C Fig**). This finding suggests that people update their current prior more after experiencing a higher effort, but not as a function of having produced more or less noisy force traces on the previous trial.

To examine the contribution of motor noise to subject-wise learning, we tested whether each participant’s mean motor noise across trials was related to their learning rate estimated from the RL model (Fig 4C). There was no significant correlation between motor noise and learning rate (*r*(27) = -0.198, *P* = 0.3136) (**S5D Fig**).

Taken together, both trial-wise and subject-wise analyses revealed that in our task, effort learning is not influenced by motor noise.

## Control analysis of within-trial force adjustment (S6 Fig)

To test whether the observed volatility effects (longer adjustment RTs under higher volatility) in **Fig 5A** were driven by change trials, we repeated the same regression on adjustment RTs but excluded change trials from the analysis. Notably, this exclusion did not change the key results (**S6A Fig**). In this regression, the only difference from the main **Fig 5A** is that the main effect of noise was not significant anymore (*t*(27) = -1.537, *P* = 0.1359). All other results were consistent with those in **Fig 5A**. Specifically, the adjustment RTs remained significantly slower in the high volatility compared to low volatility environments (*t*(27) = 2.416, *P* = 0.0227). The interaction between volatility and noise remained significant (**S6A Fig:** *t*(27) = -2.644, *P* = 0.0135), showing that the effect of volatility on adjustment RTs was larger under low noise, and the effect of noise on adjustment RTs was stronger under high volatility (**S6B Fig**). Adjustment RTs were also slower when the required effort increased from a low to a high level (jumpSign, *t*(27) = 7.213, *P* < 0.0001) or when the absolute magnitude of the change was larger (absJumpSize, *t*(27) = 8.286, *P* < 0.0001). These findings suggest that the slower adjustment under higher volatility was not confounded by change trials.

Because there is more time (i.e., more trials) to learn the true required mean effort level in low volatility compared to high volatility blocks, participants may achieve higher precision and faster learning rates in correcting priors to the true effort in low volatility environments. To test this possibility, we averaged adjustment RTs separately for early and late stages of stable (low volatility) phases where the mean effort was constant, and for all trials within a constant mean effort period in high volatility phases as before. In other words, the early trials of a low-volatility stable phase were treated as comparable to all trials in a high-volatility stable phase because they matched in duration, while late stages of a low-volatility stable phase have no equivalent in a high volatility context. This is because in low volatility, required effort changed every 10-14 trials, allowing a split into the first five versus the later five trials within a constant mean effort level. By contrast, the required effort changed every 5-7 trials in high volatility, making the entire phase naturally correspond to the early stages of a low volatility block.

**S6C** **Fig** illustrates the averaged adjustment RTs in the early and late stages of low volatility blocks, as well as high volatility blocks, across both low and high noise blocks. A two-way repeated-measures ANOVA was applied to these averaged adjustment RTs. The main effects of volatility (*F*(1,27) = 23.808, *P* < 0.0001) and noise (*F*(1,27) = 7.895, *P* = 0.0091) remained significant. The interaction between volatility and noise was also significant (*F*(1,27) = 15.679, *P* < 0.0001). The Bonferroni-corrected post hoc tests revealed faster adjustments in the late stage of a low volatility block compared with the early stage of a low volatility block, and with the comparable trials (i.e., equally early) in a high volatility block in low noise blocks (all *P* < 0.0001), but not in high noise blocks (all *P* > 0.05). We repeated this analysis again without the change trials (**S6D Fig**), which did not change the results. Together, these control analyses suggest that stability during a constant mean effort level facilitates the correction of priors under low noise environments.
