# Supplementary figures and images for: Environmental uncertainty shapes human effort learning
Source: PLoS Biol. 2026 May 7;24(5):e3003791. doi: 10.1371/journal.pbio.3003791 (PMC13175491; doi:10.1371/journal.pbio.3003791)

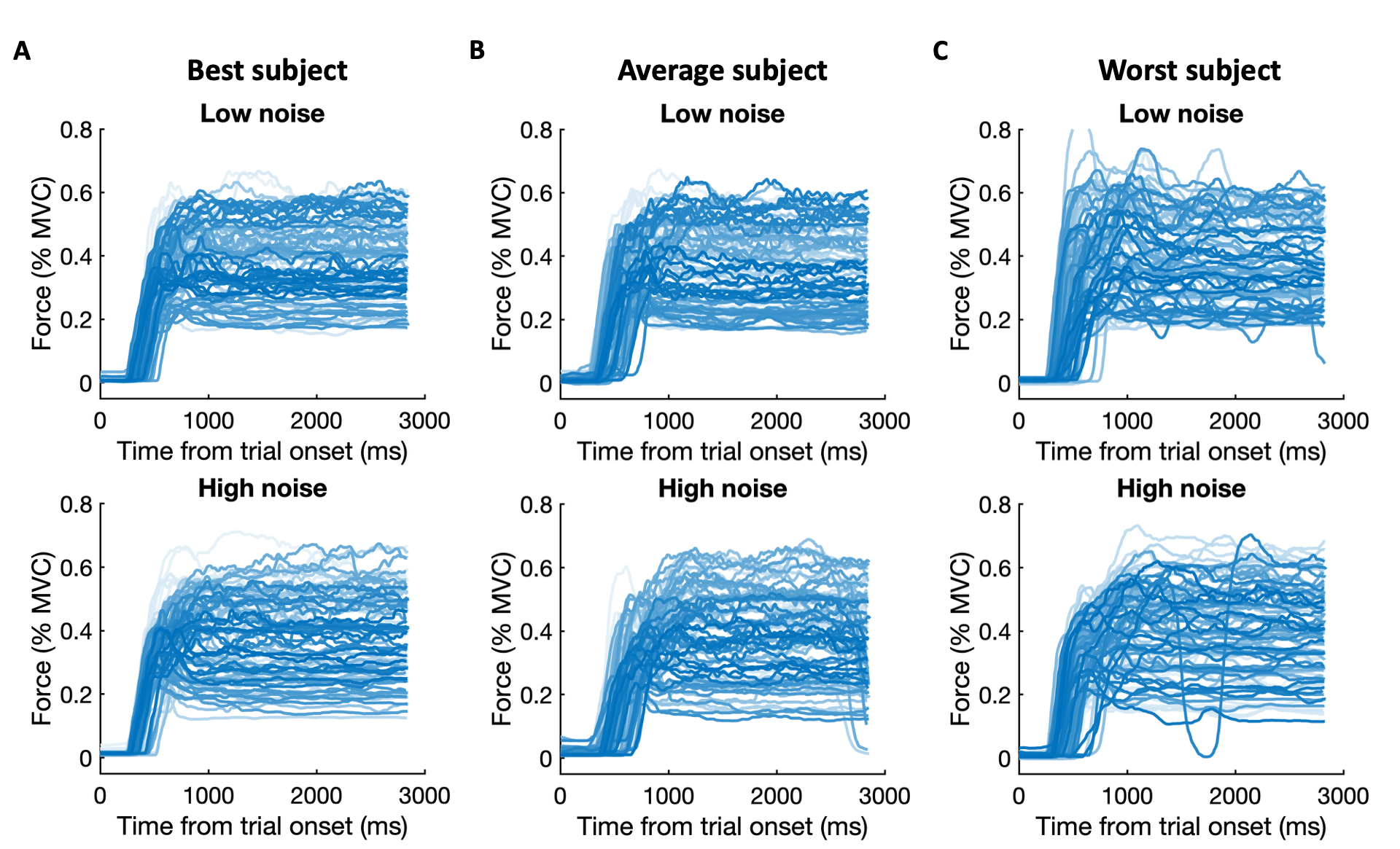

Supplement: S1 Fig — Representative participants in the categories of participants with the best (A), average (B) and worst (but not excluded) (C) overall trace quality are shown with two consecutive blocks (96 trials) in low and high noise blocks, respectively. (TIFF) [file pbio.3003791.s003.tiff]

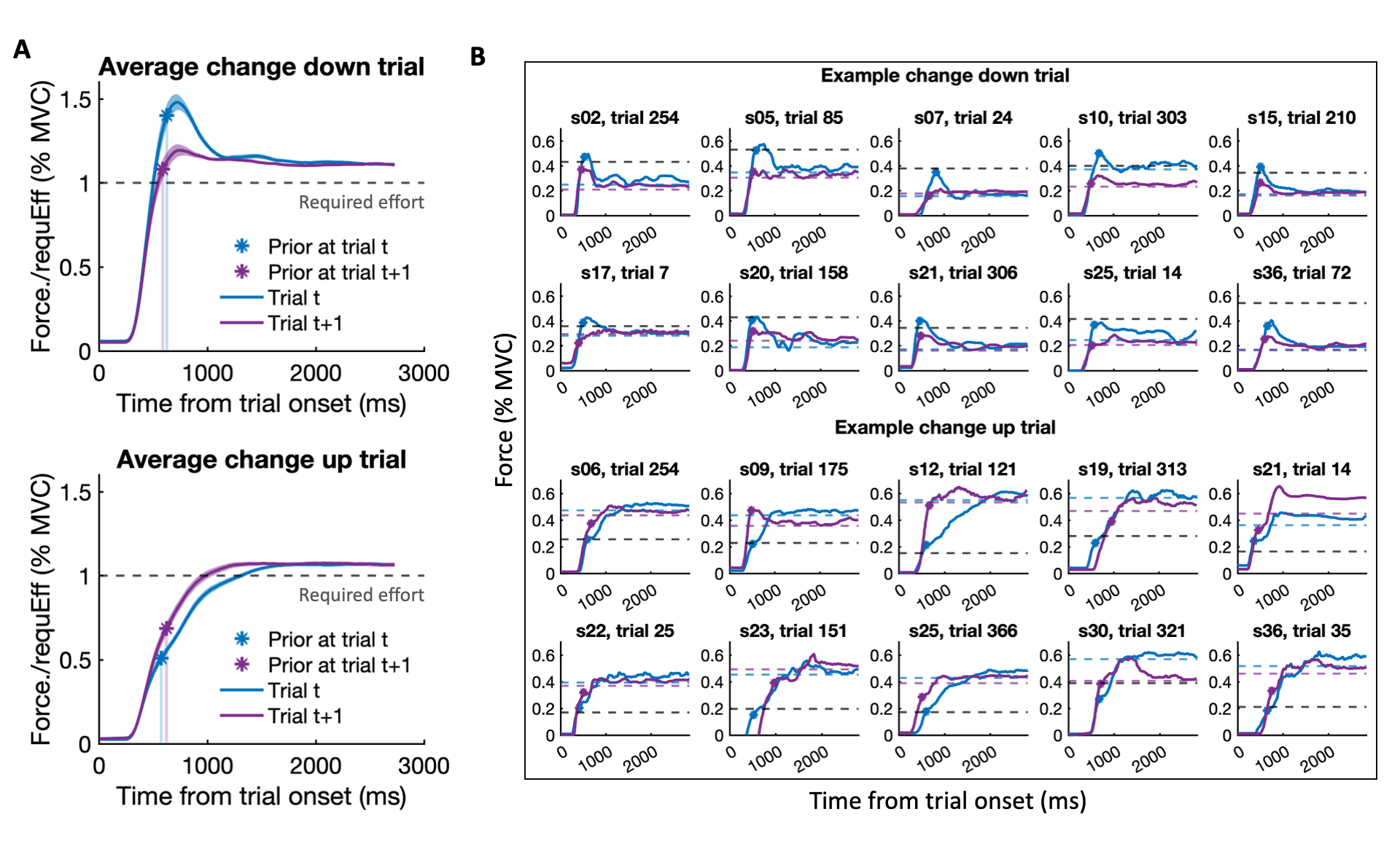

Supplement: S2 Fig — Both average force traces (A) and examples from randomly selected participants (B) showed consistent patterns: effort priors at the initial plateau overshot the required effort in change down trials and undershot the required effort in change up trials, with both errors reduced in the trial that followed the change trial (trial t + 1). In both (A) and (B), blue colour indicates the change trial t, and purple indicates the following trial t + 1. The solid line indicates the force trace. The dashed line indicates the required effort. The star indicates the effort prior. (A) The error bars in force traces indicate the standard error across participants at each time point. The vertical error bars indicate the standard error of the time point of the effort prior. (B) The black dashed line indicates the required effort in trial t-1, the blue dashed line indicates the required effort in trial t, and the purple dashed line indicates the required effort in trial t + 1. (TIFF) [file pbio.3003791.s004.tiff]

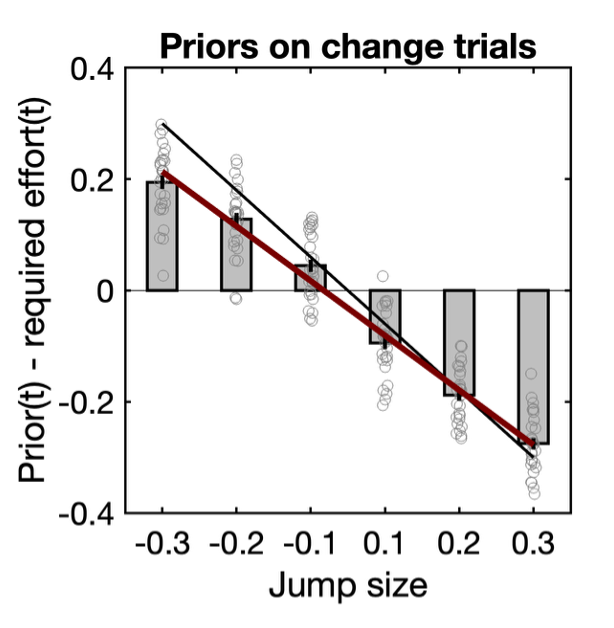

Supplement: S3 Fig — The difference between the current effort prior and current required effort is plotted for change trials with different jump sizes across all participants. The jump size is defined as the change in required effort from trial t to trial t-1 independent of noise. Given the jump could not be anticipated, if the prior merely reflects an expectation formed on previous trials, rather than sensorimotor feedback accumulated on the current trial, the error should perfectly scale with the jump size. This was indeed what was observed: the prior’s deviation from the required effort scaled with increased jump size. The observed slope (red line) was close to the ideal slope (slope = −1; black line), and deviations were smaller for upwards change trials. This shows that any influences of immediate sensorimotor feedback on our estimate of prior were minimal. All data used to generate this plot can be found in S1 Data. (TIFF) [file pbio.3003791.s005.tiff]

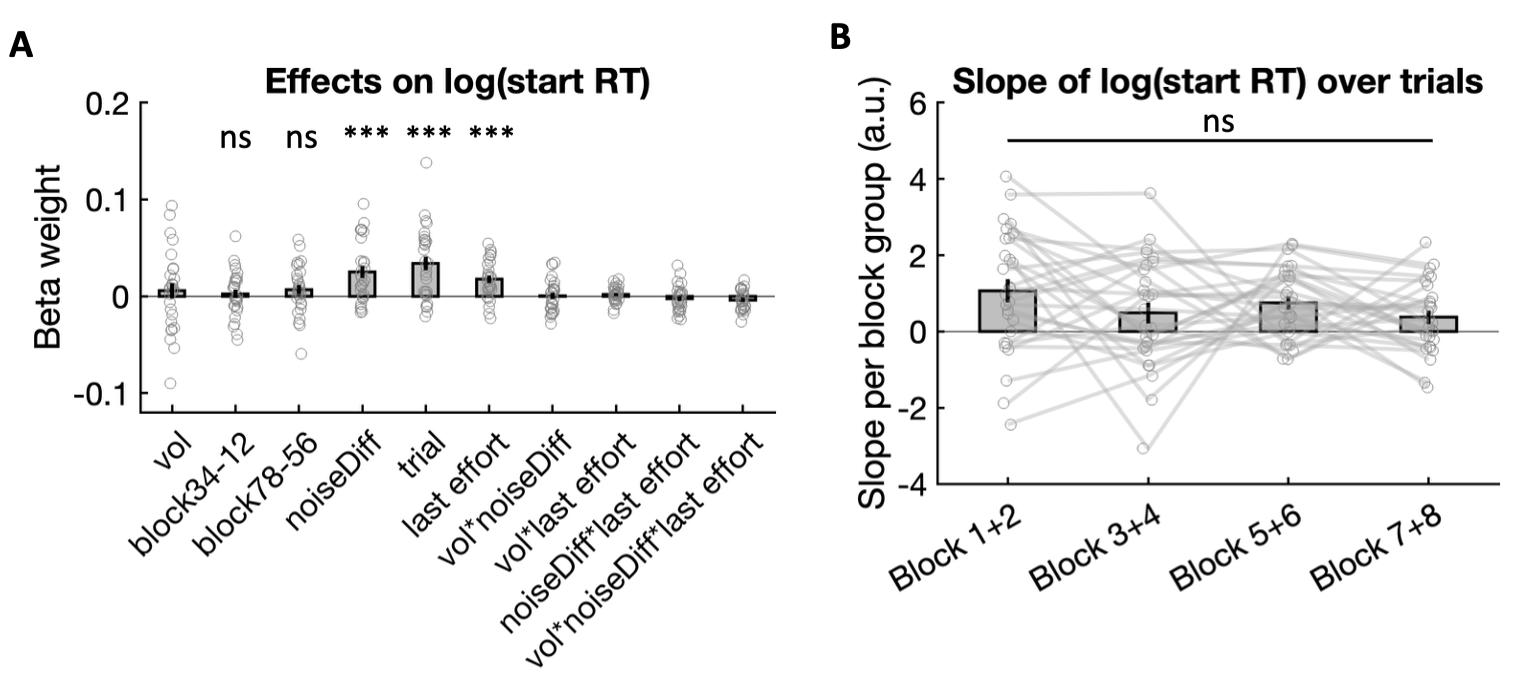

Supplement: S4 Fig — (A) Group means estimates derived from a linear regression show the effect of block-wise noise difference, trial number and last effort on start RT. Start RT did not differ between the first two block groups (block 1 + 2 versus block 3 + 4; all low noise blocks) or the last two block groups (block 5 + 6 versus block 7 + 8; all high noise blocks). Thus, the increase of start RTs over blocks is unlikely to be driven by long-term fatigue. Stars indicate significance from two-tailed one-sample t-tests on the obtained parameter estimates. (B) Slopes were fitted to start RTs over trials (trial 1–96) within each block group and slope estimates are shown here across block groups. This confirmed that the slowing of start RTs with progression through a block group was similar across all block groups of the task. The rate of increase in start RTs did not increase with time across block groups, supporting the interpretation that noise effects observed in Fig 3 are unlikely to be confounded by long-term fatigue. The error bar indicates the standard error. *** P < 0.001, ns P > 0.05. All data used to generate this plot can be found in S1 Data. (TIFF) [file pbio.3003791.s006.tiff]

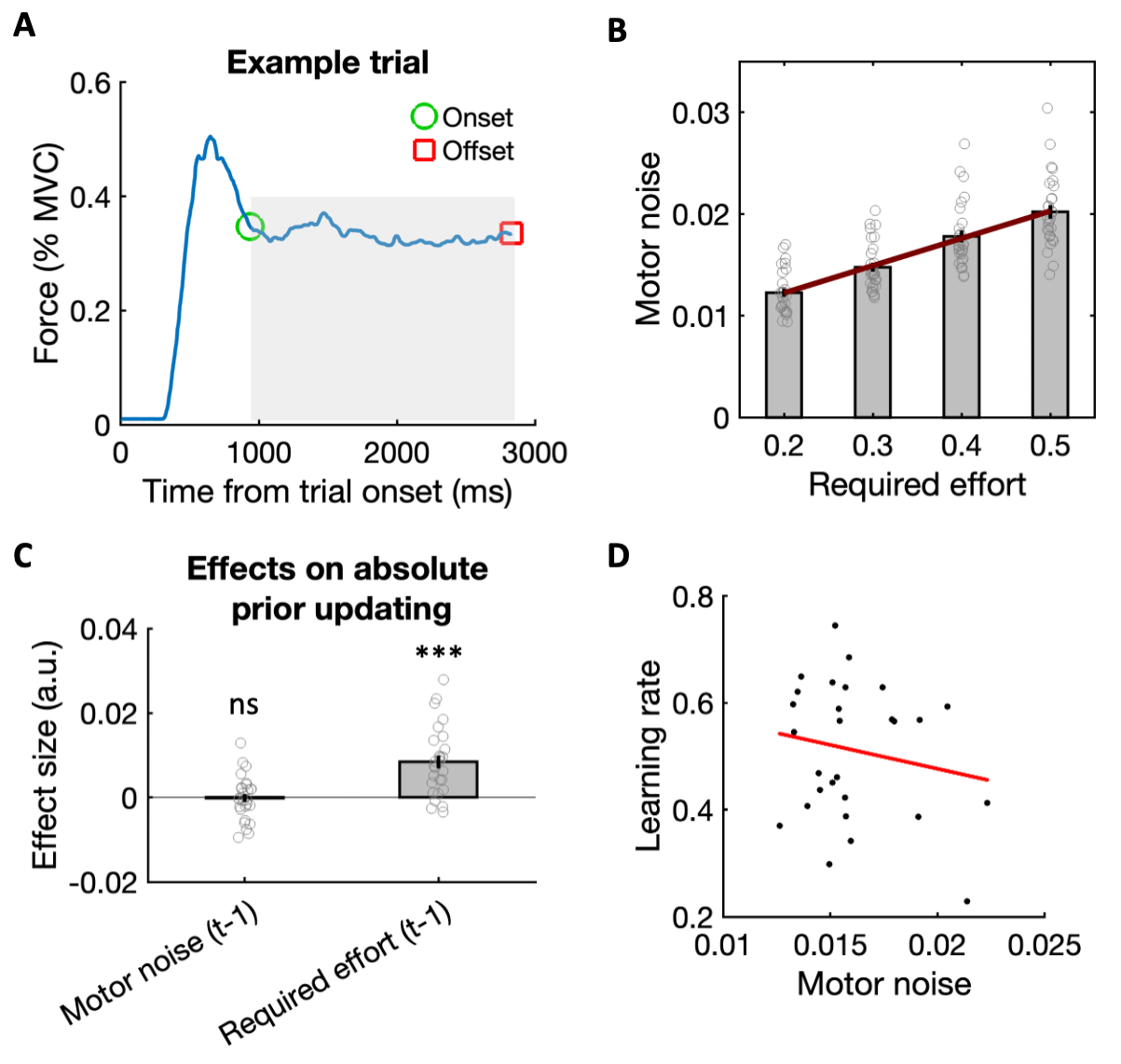

Supplement: S5 Fig — (A) An example force trace illustrates the definition of onset (green circle) and offset (red square) of the stable period (grey shading). The standard deviation of the force trace within this stable period was extracted as a measure of motor noise for each trial. (B) To validate our measure of motor noise before examining relationships with learning, we tested whether motor noise scales with required effort. The significant slope (red line) shows that motor noise increased with higher effort. (C) Group means estimates from a regression show the effects of the previous trial’s required effort and motor noise on absolute prior updating. There was no evidence to suggest that motor noise contributed to trial-by-trial learning. (D) Subject-wise correlation between motor noise and learning rate coefficients from the reinforcement-learning model. Again, there was no evidence for a relationship between motor noise and learning across participants. All data used to generate this plot can be found in S1 Data. (TIFF) [file pbio.3003791.s007.tiff]

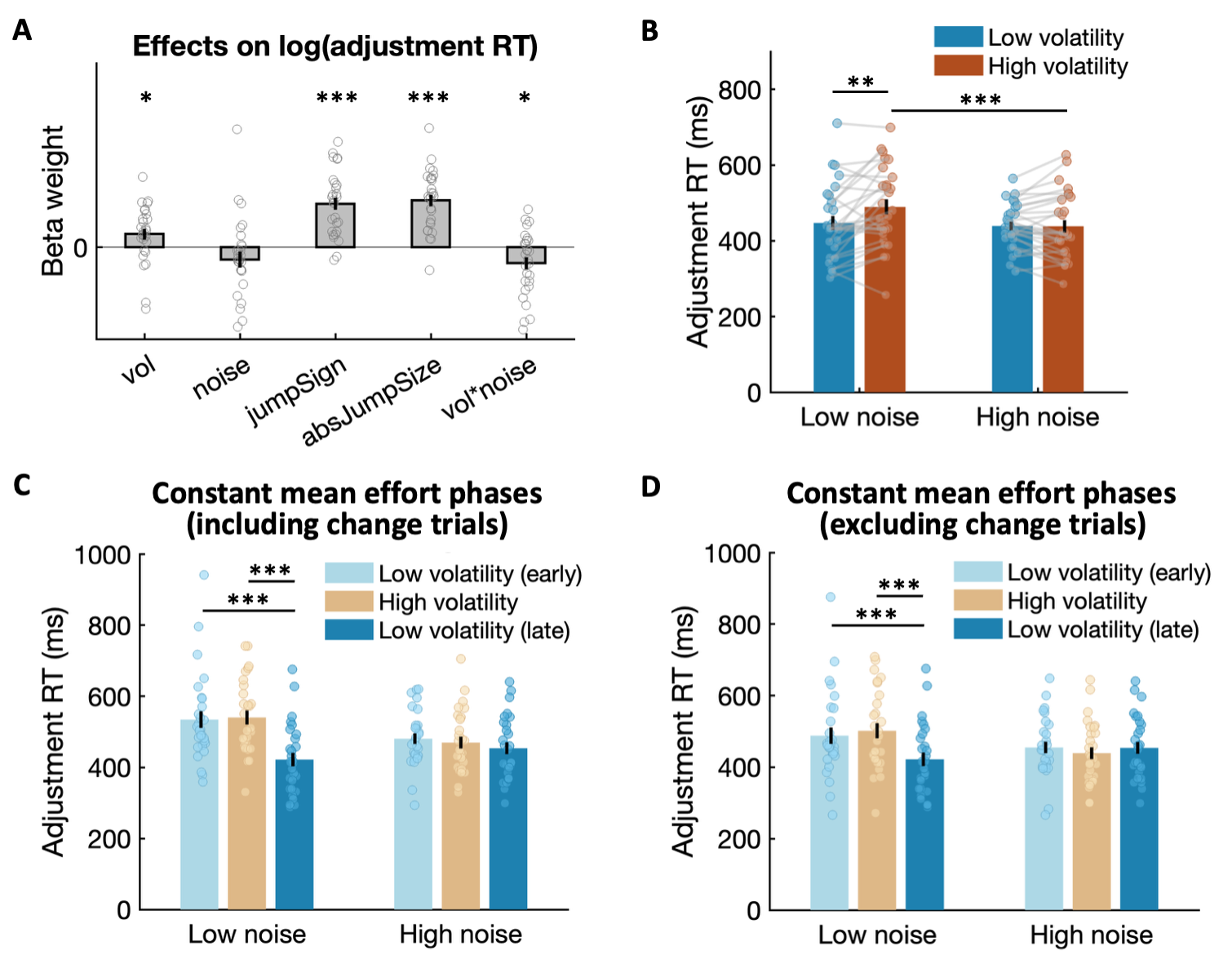

Supplement: S6 Fig — (A) Group means estimates from a regression equivalent to that reported in Fig 5B, this time after exclusion of change trials. Our key results replicated here: adjustment RTs were faster under higher noise but slower under higher volatility environments. Also, trial-by-trial changes in effort—the direction and absolute magnitude of jump both slowed down adjustment RTs. The only difference from the main Fig 5A is that the current regression showed a relatively weaker interaction (i.e., smaller t value and larger p value) between volatility and noise environments. (B) Adjustment RTs in high and low volatility environments, across low and high noise environments, after exclusion of change trials. This replicates the interaction between noise and volatility as in Fig 5B: in low noise environments, adjustment RTs were slower in high volatility compared to low volatility; in high volatility environments, adjustment RTs were faster in high noise compared to low noise environments. The only difference from the main Fig 5B is that the current interaction showed relatively weaker (i.e., smaller t value and larger p value) volatility effect (i.e., slower adjustments under higher volatility) in low noise environments. (C) To test whether stability after mean effort switches leads to faster adjustment RTs, we compared adjustment RTs between early and late stages within a constant mean effort level. To make different stages within each constant mean effort level comparable in different volatility blocks, we considered the first five trials as an early stage in low volatility, and the later five trials as a late stage in low volatility, as well as the first five trials as an entire phase in high volatility (equivalent to the early stage of a low volatility block). This is because the required effort changes every 10–14 trials in low volatility and the required effort changes every 5–7 trials, which making the entire phase in a high volatility block correspond to the early stage of [file pbio.3003791.s008.tiff]
