# Supplementary material for: Environmental uncertainty shapes human effort learning
Source: PLoS Biol. 2026 May 7;24(5):e3003791. doi: 10.1371/journal.pbio.3003791 (PMC13175491; doi:10.1371/journal.pbio.3003791)
Supplement: Attachment — Submitted filename: response letter_rb_2026-03-26.docx [file pbio.3003791.s009.docx]

We sincerely appreciate the time and effort that all the editors and reviewers have devoted to review our manuscript. Their thoughtful comments have greatly helped us improve the clarity and overall quality of our work. We have carefully revised the manuscript in response to these suggestions. All changes are highlighted in blue in the revised manuscript. Below, we provide detailed responses to each reviewer’s comments. The reviewers’ comments are shown in **bold**, and our responses are shown in standard font. Revised sections of the manuscript are highlighted in blue.

**Reviewer #1 (Nils B. Kroemer, signed his report): In their submission "Environmental Uncertainty Shapes Human Effort Learning", Bi et al. present an innovative approach to estimate the effects of uncertainty on human effort learning using a novel task design. In my opinion, this is already a strong and convincing contribution, and I have only minor suggestions to potentially further improve the submission. Strengths of the paper include the use of a sound and elegant task design, the clear description of the rationale and the methods, the high quality of the data visualization, and the overall quality of the writing and reporting of the findings. I think this is one of the best papers I have reviewed this year, and I would like to commend the authors for this relevant contribution to the effort learning literature.**

Response: Thank you very much for your positive assessment of our manuscript. We are pleased to hear that you enjoyed reading it and found it interesting. Thank you so much for your time and thoughtful comments.

**Minor issues**

**1. The differences in learning-related adjustments between noise and volatility are very interesting, including the difference to reward learning. However, unless I missed it, I did not see whether the authors analyzed correlations between the estimated adjustments to noise or volatility at the level of the individuals. In other words, is someone who is more likely to learn from noise also more likely to learn from volatility (even if the directions are opposite) or are those independent aspects of effort learning? Likewise, I was wondering how independent the different estimates of the learning process are (e.g., force initiation or adjustment RT). These additional analyses may strengthen the interpretation of the findings.**

**Response:** We thank the reviewer for suggesting these additional analyses.

To address whether the sensitivity to volatility and noise were related across individuals, we computed the correlation between the effects of volatility and noise separately for **learning rate** and **adjustment RTs**. We focused on these two measures because both were affected by volatility and noise in our main findings.

We did not observe significant correlations for either measure (p=0.36 and p=0.61). We have now included these additional analyses in the revised manuscript (lines 327-332, lines 371-375), as follows:

“To examine whether effort updates to volatility and noise were related, we computed the correlation between individual changes in log-transformed learning rate from low to high volatility and the corresponding changes from low to high noise. We did not observe a significant correlation between these learning rate changes (r(27) = -0.179, P = 0.3625), suggesting that sensitivity to volatility and noise reflects independent aspects of effort learning. “

“To examine whether within-trial adjustments to volatility and noise were related, we computed the correlation between individual changes in log-transformed adjustment RTs from low to high volatility and the corresponding changes from low to high noise. We did not observe a significant correlation between adjustment RT changes (r(27) = -0.098, P = 0.6177), suggesting that sensitivity to volatility and noise reflects independent aspects of within-trial force adjustment.”

To address the reviewer’s second question regarding the independence of different learning-related processes, we computed the correlation between force initiation and force adjustment. Specifically, for each participant, we computed the correlation between log-transformed start RT and log-transformed adjustment RT across all trials, and then tested the Fisher z-transformed correlation coefficients against zero using a one-sample t-test.

We did not observe a significant effect (indicative of consistently non-zero correlations) across individuals. We have now added this analysis in the revised manuscript (lines 197-201), as follows:

“To examine whether start RT and adjustment RT were independent, we computed the correlation between log-transformed start RT and log-transformed adjustment RT across trials for each participant and then tested the Fisher-z-transformed correlation coefficients against zero using a one-sample t-test. We did not observe a significant effect (mean r = 0.016, t(27) = 1.167, P = 0.2533), suggesting that force initiation and force adjustment reflect independent learning-related processes.”

**2. Although the differences between the pattern of effort vs. reward learning adjustments to noise are intriguing, I would be cautious to interpret this more strongly based on the evidence provided here. First, the same participants did not complete a reward learning task. Second, the effort learning task did not manipulate reward levels, which might have helped to further elucidate the link to reward-related computations. I would suggest stating more clearly that the study cannot fully answer this question and that future research should follow-up on this potential discrepancy.**

**Response:** We agree with the reviewer that our task design does not allow a direct comparison between effort learning and reward learning in response to volatility or noise. As suggested, we have clarified this limitation in the Discussion (lines 542-544), as follows:

“The design of this study also leaves several open questions for future research. First, because reward learning was not directly manipulated, conclusions about cross-domain differences between effort and reward learning remain tentative.”

**3. The number of excluded participants is quite high, although the stated criteria seem reasonable to me. Please specify if the exclusion criteria were defined a priori and how generalizable the findings are in this sample. Are there participants where the model does not provide a good fit of the behavioral data?**

**Response:** We acknowledge that the number of excluded participants is relatively high. All exclusion criteria were defined a *priori* to ensure that the learning-related measures could be estimated reliably. These criteria were based solely on data quality considerations (e.g., insufficient quality of force traces or inadequate number of valid samples). In other words, no exclusions were made based on statistical outcomes or goodness of model fitting.

To clarify this point, we first show two representative participants (s13 and s25) with average overall trace quality as a reference. For each trial, the force trace was rescaled to the required force level. As shown below, these participants reached the required effort and maintained the force for at least 1000-1500 ms in all trials. Across trials, the deviation between the produced force and the required effort during the stable period was small, reflecting accurate force adjustments. In addition, the force traces were smooth overall and exhibited minimal fluctuations.

Next, we provide force traces corresponding to each exclusion criterion. In total, this excluded seven participants, but some fulfilled more than one criterion and are only shown once below.

**Criterion 1**: Four participants reached and maintained the required effort for at least 1000 ms in less than 80% of trials (mean duration of force within effort range was 1600 ± 233 ms across all 35 participants).

**Criterion 2**: One additional participant (as well as s31 above) did not follow the task instruction to adjust effort to the required levels. As can be seen below, s03 pressed at a random force on early trials (light blue: colour darkness scales with trial number).

**Criterion 3**: Two additional participants (as well as s08 above) exhibited unusually variable and noisy force traces such that the percentage of trials in which a steady state was detected was lower than 80%. In other words, the noisy trace made it difficult to detect a stable period even though the effort may have successfully surpassed the required effort.

As shown in these examples, the excluded datasets did not provide reliable estimation of learning or adjustment over time, supporting the necessity of these exclusions. We hope the reviewer agrees that these conclusions were justified. We would be happy to show these traces in the supplementary information if you felt that this would strengthen the methodological rigour of our manuscript. For now, we decided this might be too much detail and have left things as they are.

**4. Relatedly, were any alternative models explored (and compared to the final model)?**

**Response:** We thank the reviewer for raising this important point. We assume the reviewer is referring to the reinforcement learning (RL) model presented in Fig.4C. In this study, we did not formally explore or compare alternative models. Our primary goal was to characterize core updating processes and to test whether these differed across environmental statistics (volatility and noise), which can be captured by the standard PE-based model. Importantly, this model was sufficient to account for the key effects reported in the manuscript.

We agree that other models could be considered. For example, models allowing asymmetric learning rates depending on the sign of the effort prediction error may provide additional insights. A systematic comparison of such models would be valuable but would go beyond the central aims of the present study.

To clarify this point, we have expanded the Discussion to consider potential alternative models (lines 470-473), as follows:

“While we focused on this standard PE-based model to characterize basic updating processes, other alternative models (e.g., asymmetric learning rates that depend on the sign of prediction errors) may provide additional insights and could be explored in future work.”

In addition, we will make the data and analysis scripts openly available to facilitate future exploration and comparison of alternative models.

**5. Could the authors comment on the subjective experience of participants? I agree that the conceptual link to energy metabolism is of interest, but a related question is whether the participants are consciously aware of their behavioral adjustments to noise and volatility. Did the authors ask any questions about the strategy of the participants and/or their subjective level of exertion?**

**Response:** We thank the reviewer for this thoughtful comment regarding the subjective experience of participants. It is possible that the participants became aware of differences in volatility and noise as the experiment progressed. However, concepts related to noise, volatility or environmental changes were not mentioned in the task instructions prior to the experiment. Unfortunately, we did not collect self-report measures regarding participants’ strategy or their feelings of effort during the task.

To address this and related comments from Reviewer 3, we have now included these points as limitations in the Discussion section of the revised manuscript (lines 544-551), as follows:

“Second, we did not mention concepts related to environmental changes (i.e., volatility or noise) in the task instructions, nor did we assess participants’ conscious awareness of their adjustments to these environmental statistics, making it difficult to determine whether these adaptations occurred explicitly or implicitly. Finally, we did not collect subjective reports of effort exertion. Previous work has shown that greater motor variability in exertion is associated with increased perceived effort (52–55), and future studies could examine how motor variability contributes to the subjective experience of effort and thereby influences motivational regulation.”

**6. In Figure 1, the caption for the high noise trials is misspelled (as how). Moreover, I found the visualization of either high or low noise trials a bit difficult to understand initially because I thought that they alternated from trial to trial. Maybe this can be improved.**

**Response:** We thank the reviewer for carefully reviewing our manuscript. As suggested, we have corrected the typo in Figure 1C and revised the figure by presenting separate plots for the low and high noise blocks. We believe this revision clarifies the task structure and avoids the impression that noise conditions alternated from trial to trial. We have pasted the new figure below.

Thanks again for all your helpful suggestions and for you careful reading of our manuscript. We hope you will find that our manuscript has improved as a result of this revision.

**Reviewer #2: The manuscript by Bi and colleagues investigates how humans learn required motor effort during decision making. Most studies investigating effort-based decision making explicitly indicate the required effort to obtain a reward to participants at the outset of each trial. Here, the authors investigate how this effort is learnt from trial and error, and how this learning is shaped by environmental statistics. They focus on two properties of the environment, noise (irreducible or expected uncertainty) and volatility (or unexpected uncertainty), that have been frequently investigated in the reward-based domain. The authors focus on three stages and how they are influenced by task parameters: force initiation, prior (reflecting participants' estimate of the required effort) and adjustment in response to sensory feedback. Overall, I think this is an interesting study that is carefully designed and analyzed, the analyses appear straightforward and well described, and the data support the authors' conclusions. The manuscript is well written and easy to read. I actually only have a few rather minor comments:**

Response: Thank you very much for your positive feedback and for your helpful suggestions and comments. We are pleased to hear that you found the manuscript overall well designed and easy to follow. We have addressed all comments in the below.

**1. The definition of the "prior" appeared somewhat surprising to me. By the authors' reasoning, this is thought to reflect participants' estimate of how much effort will be required on the current trial. Here, the prior is defined as the point at which, after force initiation, the force trace plateaus. I would argue that, at this stage, people already integrate the sensorimotor feedback they receive online with their prior estimate of required effort. I would not be too adamant about this point, as the data clearly indicate sensitivity of the prior across trials (Fig. 2F), but this does not preclude that this measure also included variance relate to online sensorimotor control.**

**Response:** We thank the reviewer for this thoughtful comment regarding the conceptual purity of our measure of the effort prior (i.e., the initial plateau of the force trace). We agree that some degree of rapid sensorimotor feedback integration could occur during this early stage. However, such integration is likely limited given the relatively short duration of the prior stage (M = 230.32 ms, SD = 25.11 ms) compared to the subsequent adjustment stage (M = 475.37 ms, SD = 76.58), where most feedback-driven adjustments are likely to occur.

In an ideal case, where no feedback is integrated, the deviation of the prior on trial t from the required effort on trial t would perfectly scale with jump size, i.e. the unpredictable difference in required effort level between trial *t* and trial *t-1*. If this was the case, the prior would reflect only previous expectations but not current requirements. This can be most conservatively investigated at change trials, where the prior is off and should be updated rapidly, and where sensorimotor feedback should most clearly indicate this deviation.

To quantify the influence of sensorimotor feedback on the prior, we therefore examined how the difference between prior on trial *t* and the effort requirement on trial *t* varied with jump size for change trials (i.e., difference between required effort level from trial *t-1* to trial *t*). We found that the difference between prior and current effort scaled with jump size (see the figure below). Moreover, the observed slope was -0.778 (red line), slightly flatter than the ideal slope (i.e., slope = -1; black line). This deviation could indicate a small influence of sensorimotor feedback. We noticed that this deviation was more pronounced for downwards and smaller for upwards change trials, potentially reflecting an energy-conserving strategy (for negative down-ward changes, naturally the prior was at a higher effort level). Thus, these results suggest that our estimated prior primarily reflects expectation-based beliefs rather than feedback-driven integration.

**Figure S3. The estimated prior is not driven by sensorimotor feedback.** The difference between the current effort prior and current required effort is plotted for change trials with different jump sizes across all participants. The jump size is defined as the change in required effort from trial t to trial t-1 independent of noise. Given the jump could not be anticipated, if the prior merely reflects an expectation formed on previous trials, rather than sensorimotor feedback accumulated on the current trial, the error should perfectly scale with the jump size. This was indeed what was observed: the prior’s deviation from the required effort scaled with increased jump size. The observed slope (red line) was close to the ideal slope (slope = -1; black line), and deviations were smaller for upwards change trials. This shows that any influences of immediate sensorimotor feedback on our estimate of prior were minimal.

We acknowledge that it is not possible to completely exclude the influence of rapid feedback integration. To address the reviewer’s concern, we have now included this control analysis in the Supplementary information (Supplementary Fig S3) and added the relevant description to the Results section (lines 226-229), as follows:

“The effort prior was defined at the initial plateau, which raises the possibility that sensorimotor feedback may have been integrated into the estimated prior. However, control analyses revealed only a minimal influence of online feedback at this early stage (prior duration: M = 230.32 ms, SD = 25.11 ms; Supplementary Fig S3).”

We further clarified this point in the Discussion section (lines 421-423), as follows:

“Although we cannot entirely exclude the possibility that sensorimotor feedback contributed to the estimated prior, our control analyses suggest that such influence was minimal.”

**2. In the abstract, it is mentioned that effort learning is investigated "across different stages of the effort production process (e.g., initiation, prior, adjustment)" - I guess, at this stage, the reader probably cannot have a detailed idea what these phases are - in particular, "prior" to me sounded like a statistical quantity (a prior estimate/knowledge/probability), not a temporal stage of a trial. Perhaps either omit - or add a short sentence describing this?**

**Response:** We agree with the reviewer that these stages should be clarified when first introduced in the Abstract. We have revised the description of these stages in the Abstract (lines 43-44), as follows:

“across different stages of the effort production process (e.g., initiation of effort production, effort expectation, error-driven adjustment)”

**3. The regression results in 4B show an effect of past effort predominantly from trials t-1 and t-2 on the current prior, with a clear effect of noise, but not volatility. This is strongest on t-1. This finding seems to contrast with the learning rate coefficients in 4C, which are affected by both noise and volatility. What is the reason for this discrepancy?**

**Response:** We thank the reviewer for pointing out the difference between regression-based analysis (Fig. 4B) and the reinforcement learning (RL) model results (Fig. 4C). We apologize that this was not clarified in the original manuscript. Although the two approaches address updating processes, they are mathematically closely related but not strictly identical.

In the RL model, **expected** **effort** $\boldsymbol{Q}_{\boldsymbol{t}}$ is updated based on the previous effort expectations $\boldsymbol{Q}_{\boldsymbol{t-1}}$ and last experienced effort $\boldsymbol{e}_{\boldsymbol{t-1}}$, according to the formula:

$Q_{t}={\alpha\cdot e}_{t-1}+{(1-\alpha)\cdot Q}_{t-1}$,

where $e_{t-1}$is the required effort and *α* is the learning rate. The formula in the RL model can be expanded by substituting $Q_{t-1}$ into the equation:

$Q_{t}={\alpha\cdot e}_{t-1}+ \left( 1-\alpha\right)\cdot{[\alpha\cdot e}_{t-2}+{\left( 1-\alpha\right)\cdot Q}_{t-2}]$

$={\alpha\cdot e}_{t-1}+\alpha\cdot\left( 1-\alpha\right)\cdot e_{t-2}+{\left( 1-\alpha\right)^{2}\cdot Q}_{t-2},$

By repeatedly substituting $Q_{t-2}, Q_{t-3}, etc$. The learning rule in the RL model is equivalent to:

$Q_{t} = \sum_{\tau=1}^{t} \alpha\cdot(1-\alpha)^{\tau-1}{\cdot e}_{t-\tau}$.

Thus, the RL model integrates information across the full effort history with exponentially decaying weights. This means the shape of the decay is predetermined as an exponential shape and the learning rate just captures its decay rate.

In contrast, the regression model considers only the three most recent trials.

$$P_{t}={\beta_{1}\cdot e}_{t-1}+\beta_{2}\cdot e_{t-2}+{\beta_{3}\cdot e}_{t-3}$$

This regression model can broadly be viewed as a truncated RL model, which captures only a limited recent history, but which allows a more flexible shape because the dependencies between the betas is not predetermined by a function.

$Q_{t}= \sum_{\tau=1}^{3} \alpha\cdot(1-\alpha)^{\tau-1}.$

Thus, although both approaches characterise how past effort influences current expectations, they differ in how the learning history is modelled and fitted. The RL model integrates information across the full history with decaying weights determined by a single free parameter (*α*), resulting in a fixed kernel shape. In contrast, the regression model restricts estimation to three lags and fits a separate parameter for each lag ($\beta_{1}$,$\beta_{2},\beta_{3})$, which allows for a more flexible kernel. These differences in temporal integration and model fitting likely account for the discrepancy in volatility effects observed between the two analyses.

To clarify this point, we have now added a brief explanation to the Discussion section (lines 465-470), as follows:

“Although the regression-based analysis and the PE-based model both characterize updating processes, they are not mathematically identical. The regression model captures the influences of a limited recent history (i.e., the past three trials with independent weights), whereas the RL model evaluates updating over the full effort history (with a fixed kernel shape). Differences in the observed volatility effect across the two approaches likely reflect differences in temporal integration.”

**4. Regression models investigating start/adjustment RT: do these RT measures also show the typical non-Gaussian (skewed) distribution? If so, was log(RT) used for the analyses?**

**Response:** Thank you for raising this important point. Both start RTs and adjustment RTs exhibited mildly positively skewed distributions. In the original manuscript, all regression analyses were conducted on raw RTs. As suggested, we have now re-fitted the regression models using log-transformed RTs. All the effects remained when we repeated the regression using log-transformed RTs. For visualization purposes, we continue to show mean raw RTs in Fig. 3B and Fig. 5B to preserve interpretability.

We have clarified this point in the Methods and updated the relevant figures (Fig. 3A and Fig. 5A; supplementary Fig. S3A-S3B and Fig. S4A) in the revised manuscript.

**5. Maybe I've missed this, but how were people incentivized/rewarded?**

**Response:** We apologized that this was not clearly described. We have now clarified this information in the Methods section (lines 588-590), as follows:

“The longer participants succeeded at keeping the blue fluid within the target range, the more reward they would receive at the end of the experiment (bonus between £0 and £3 scaled directly with the time spent within the 100-120% target zone)”.

Thanks again for all your helpful suggestions and for you careful reading of our manuscript. We hope you will find that our manuscript has improved as a result of this revision.

**Reviewer #3: This study investigates how healthy individuals learn to regulate effort in response to varying environmental uncertainties. Using a novel effort-learning task with a handheld dynamometer, the experimental task manipulates two forms of uncertainty: volatility by varying the number of exertion levels and noise by altering feedback from the effort-exertion cursor. The findings show that participants effectively estimate required effort by integrating prior experiences and immediate sensorimotor feedback. Importantly, the study reveals that environmental conditions significantly influence effort-learning dynamics, with high-noise environments resulting in slower initiation, weaker effort priors, and rapid within-trial adjustments; while high-volatility contexts lead to slower learning and adjustments. The results suggest that different sources of uncertainty are incorporated into internal effort estimates, providing insights into the mechanisms of effort learning. This study explores a timely question — effort learning — which has received limited investigation. The experiments are well designed and the analytical approaches used are suitable. I have some suggested analyses to strengthen the manuscript**

Response: Thank you very much for your positive assessment of our manuscript and for your very helpful suggestions. We appreciate the time that has been put into this review and have now addressed the comments raised below.

**1. The authors manipulate noise by providing visual feedback of exertion sampled from different distributions. It appears that the actual force traces illustrated in Figure S1 are quite noisy — resulting from motor variability/noise. It would be important to show that the actual motor noise on a trial is not related to learning rates. This motor noise could be another form of uncertainty that impacts learning (in addition to the volatility and 'visual' noise manipulated in the experiment). The authors should perform analyses to account for the motor variability/noise factor.**

**Response:** We thank the reviewer for suggesting control analyses to evaluate whether motor noise contributes to learning during effort production. To address this, we examined the contribution of motor noise at both the trial-wise and subject-wise levels.

We first quantified trial-wise motor noise for each participant. Motor noise was defined as the standard deviation of the force trace during the stable period. The onset (green circle) of this window was already obtained (panel A in the figure below). To determine the offset (red square), (1) we smoothed the derivative of the raw force trace (smoothing kernel: 40 samples); (2) we then computed the derivative of the smoothed force trace; (3) finally, we identified the latest time point in the force trace where both the smoothed force exceeded 0.1 and its derivative surpassed a threshold of -0.0001. The standard deviation of the force trace within this period (width of grey shading) was then used as the trial-wise estimate of motor noise.

To validate our measure of motor noise before examining relationships with learning, we tested whether motor noise scales with required effort. This is also in line with the reviewer’s suggestion further below in comment 2. Indeed, we found that the level of motor noise scaled with the level of required effort, with larger noise for higher efforts as expected (t-test on each participant’s slope: *t(*27) = 16.413, *P* < 0.0001; see also panel B below).

**Figure S5. Motor noise is not related to effort learning. (A)** An example force trace illustrates the definition of onset (green circle) and offset (red square) of the stable period (grey shading). The standard deviation of the force trace within this stable period was extracted as a measure of motor noise for each trial. **(B)** To validate our measure of motor noise before examining relationships with learning, we tested whether motor noise scales with required effort. The significant slope (red line) shows that motor noise increased with higher effort. **(C)** Group mean estimates from a regression show the effects of previous trial’s required effort and motor noise on absolute prior updating. There was no evidence to suggest that motor noise contributed to trial-by-trial learning. **(D)** Subject-wise correlation between motor noise and learning rate coefficients from the reinforcement learning model. Again, there was no evidence for a relationship between motor noise and learning across participants.

In a first analysis, to assess the contribution of motor noise to trial-wise learning, we therefore fitted a linear regression, modelling the absolute prior update in the current trial as a function of the motor noise and effort requirements in the previous trial. The results show that absolute prior updating was significantly influenced by the previous trial’s experienced effort (*t*(27) = 5.563, *P* < 0.0001), but not by motor noise (*t*(27) = -0.177, *P* = 0.8606) (panel C). This finding suggests that people update their current prior more after experiencing a higher effort, but not as a function of having produced more or less noisy force traces on the previous trial.

We also considered repeating the main regression from our manuscript with motor noise included. When we did this, this did not change any of our conclusions, and motor noise itself did not explain significant variance (P > 0.05). However, we are not sure whether this analysis makes perfect sense because we would expect motor noise to change the absolute size of the update, but not the signed update or prior itself which is what the analyses in our manuscript focus on.

In a second analysis, to examine the contribution of motor noise to subject-wise learning, we tested whether each participant’s mean motor noise across trials was related to their learning rate estimated from the RL model (Fig. 4C). There was no significant correlation between motor noise and learning rate (*r*(27) = -0.198, *P* = 0.3136) (panel D).

We have now included these additional methods and results in the supplementary information (supplementary figure S5), and we refer to them in the Results of the revised manuscript (lines 332-333). Please also note that the analyses above are based on our understanding/interpretation of this comment. Please let us know if you had something else in mind.

“We also examined whether learning is influenced by motor noise, but our control analyses found no evidence for this in our task (Supplementary **Fig S5**).”

**2. Another issue that should be considered is the idea of signal-dependent noise — exertion variability increases with increasing exertion level (Jones et al., 2002; Haris and Wolpert, 1998). While the authors control visual feedback in the experiment's noise condition, there is still motor noise that scales with exertion levels. It would be important to control for the motor noise to evaluate how this form of uncertainty might contribute to learning, keeping in mind that it will scale with the levels of exertion.**

**Response:** We thank the reviewer for raising this important point. Following the reviewer’s suggestion, we examined this relationship and found that motor noise does indeed scale with the level of required effort in our data (panel B in the figure above), as expected. We have included this additional result and figure in the supplementary information (Supplementary **Fig S5**).

When evaluating the contribution of motor noise to learning (comment 1), we indeed controlled for required effort as a potential confound in the regression analysis. When we did this, motor noise itself did not explain significant variance (P > 0.05). We hope that this addresses the reviewer’s point but as above, we would be happy to know if you had a different analysis in mind.

**3. Related to this signal-dependent noise idea, several studies of effort-based judgements have shown that variability in exertion is related to increased feelings of effort (Salimpour and Shadmehr, JNeuro, 2014; Salimpour, Mari, Shadmehr, JNeuro, 2015; Hu et al, JNeuro, 2022; Padmanabhan et al, npj Parkinsons, 2023;), and the authors should discuss their findings in the context of this previous work.**

**Response:** We thank the reviewer for pointing out these important studies. We have now cited these papers and discussed their findings in the Discussion section (lines 547-551) as follows:

“Finally, we did not collect subjective reports of effort exertion. Previous work has shown that greater motor variability in exertion is associated with increased perceived effort (52–55). In our data, motor variability in the stable phase is greater at higher effort levels, suggesting that these levels might subjectively feel even harder. However, we did not find that motor learning directly impacted effort learning. Future studies could examine how motor variability contributes to the subjective experience of effort and thereby influences motivational regulation.”

Thanks again for all your helpful suggestions and for you careful reading of our manuscript. We hope you will find that our manuscript has improved as a result of this revision.
